# Use of immunohistochemical biomarkers as independent predictor of neoplastic progression in Barrett's oesophagus surveillance: A systematic review and meta-analysis

**DOI:** 10.1371/journal.pone.0186305

**Published:** 2017-10-23

**Authors:** Vincent T. Janmaat, Sophie H. van Olphen, Katharina E. Biermann, Leendert H. J. Looijenga, Marco B. Bruno, Manon C. W. Spaander

**Affiliations:** 1 Department of Gastroenterology and Hepatology, Erasmus University Medical Center Rotterdam, Rotterdam, The Netherlands; 2 Department of Pathology, Erasmus University Medical Center Rotterdam, Rotterdam, The Netherlands; University Hospital Llandough, UNITED KINGDOM

## Abstract

**Introduction:**

The low incidence of oesophageal adenocarcinoma (EAC) in Barrett's oesophagus (BE) patients reinforces the need for risk stratification tools to make BE surveillance more effective. Therefore, we have undertaken a systematic review and meta-analysis of published studies on immunohistochemical (IHC) biomarkers in BE to determine the value of IHC biomarkers as neoplastic predictors in BE surveillance.

**Materials and methods:**

We searched MEDLINE, EMBASE, Web of Science, CENTRAL, Pubmed publisher, and Google scholar. All studies on IHC biomarkers in BE surveillance were included. ORs were extracted and meta-analyses performed with a random effects model.

**Results:**

16 different IHC biomarkers were studied in 36 studies. These studies included 425 cases and 1835 controls. A meta- analysis was performed for p53, aspergillus oryzae lectin (AOL), Cyclin A, Cyclin D and alpha-methylacyl-CoA racemase. Aberrant p53 expression was significantly associated with an increased risk of neoplastic progression with an OR of 3.18 (95% CI 1.68 to 6.03). This association was confirmed for both non-dysplastic BE and BE with low-grade dysplasia (LGD). Another promising biomarker to predict neoplastic progression was AOL, with an OR of 3.04 (95% CI 2.05 to 4.49).

**Discussion:**

Use of p53 IHC staining may improve risk stratification in BE surveillance. Aberrant p53 expression in BE patients appeared to be associated with a significantly increased risk of neoplastic progression for both non-dysplastic and LGD BE patients.

## Introduction

Development of oesophageal adenocarcinoma (EAC) is related to Barrett's oesophagus (BE), a premalignant condition of the distal oesophagus. In BE, the pre-existent squamous epithelium is replaced by columnar epithelium which develops under the influence of chronic acid and bile reflux and frequently contains goblet cells [1–3]. The progression from BE to EAC is a gradual process, in which intestinal metaplasia (IM) evolves to low-grade dysplasia (LGD), high-grade dysplasia (HGD) and eventually EAC [4]. Therefore, current guidelines recommend endoscopic surveillance in BE patients to detect HGD or EAC at an early stage, with the aim to improve survival rates [5, 6]. Several studies have shown that patients diagnosed with EAC during BE surveillance have earlier staged tumors and probably better survival compared to those diagnosed after the onset of symptoms [7–10].

The estimated incidence of EAC in patients with BE was reported to be between 0.5 and 1% per year [11–14]. However, more recent population-based studies and two meta-analyses have set this risk around 0.12% to 0.38% per year [15–18]. This relatively low annual risk reinforces the need for risk stratification tools to make BE surveillance more effective. BE length, male gender, smoking, and LGD are known risk factors for progression to HGD and EAC [13, 15, 18–20]. Two large population studies confirmed that patients with LGD have an approximately five times higher risk of progression compared to patients with non-dysplastic BE [15, 18]. Thus, more intensive surveillance is recommended in BE patients with LGD [5, 6]. However, the histological diagnosis of LGD is subject to a considerable inter- and intra-observer variation, because of sample error and overlap with features of non-neoplastic regenerative changes [21–24].

Because none of the current clinical and histologic criteria are able to accurately predict which patients are likely to progress to HGD or EAC, there is an increasing interest in (molecular) biomarkers. Many immunohistochemical (IHC) biomarkers have been studied in BE progression, mainly because they can be applied to standard histological samples. In clinical practice, IHC biomarkers are relatively easily applicable compared to other techniques. Currently, the addition of p53 IHC to the histological assessment is recommended in the guideline of the British Society of Gastroenterology as it may improve the diagnostic reproducibility of a histological diagnosis of LGD [5]. The use of IHC biomarkers as independent predictor of neoplastic progression is not yet performed in routine clinical care, neither for p53, nor for other IHC biomarkers. Therefore, this study aims to provide a systematic review and meta-analyses of all retrospective case control or cohort studies and prospective cohort studies investigating IHC biomarkers as predictor of neoplastic progression in patients with BE.

## Materials and methods

This review was conducted according to the PRISMA and MOOSE guidelines (S1 MOOSE checklist, S1 PRISMA checklist) [25, 26].

### Definitions

BE was defined as columnar lined oesophagus (CLE). Neoplastic progression was defined as the development of HGD or EAC during follow up. Patients with neoplastic progression were classified as cases and patients without neoplastic progression as controls.

### Data sources and searches

Records were identified by searching the following electronic databases: 1. EMBASE, 2. MEDLINE, 3. Web of Science, 4. CENTRAL, 5. PubMed Publisher, 6. Google scholar until 09-12-2016 (S1 Search). The search strategy was constructed by applying a sensitivity maximizing approach. A combination of MESH subject headings and text words were used related to IHC markers for progression in patients with BE. The search was confined to English language publications. Conference abstracts indexed in Embase from the years 2014–2016 were included in order to be able to include new and unpublished papers.

### Study selection

**Fig 1:**
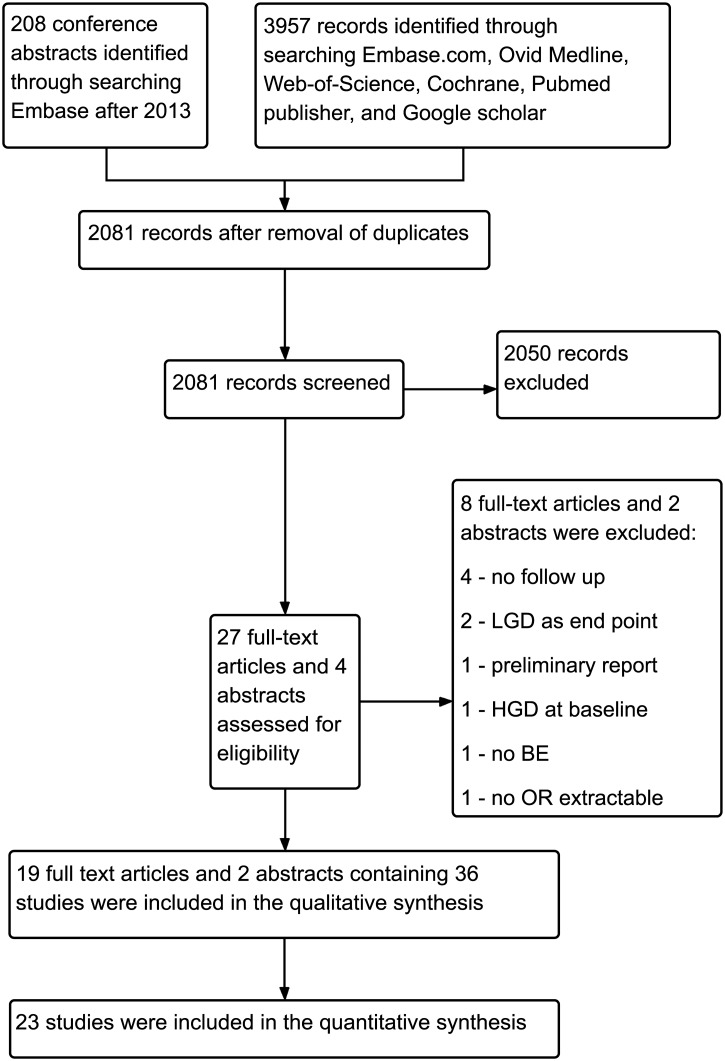
Flow chart of the study.

Search results were combined and duplicates removed. Every article was screened on title and abstract level for relevance by a single author (SvO or VJ). Articles were reviewed full text by the same two independent authors and included if they met the following criteria: (1) association between IHC biomarker expression on formalin fixed paraffin embedded material and risk of neoplastic progression was assessed; (2) a cohort or case-control study design; (3) patients with known or newly diagnosed BE with or without LGD at baseline; (4) patients defined as cases had to have progressed to either HGD or EAC during follow-up; (5) mean follow-up of at least one year from the time of initial BE diagnosis; (6) the possibility to extract an OR. Studies were excluded if: (1) BE cohorts included patients with HGD at baseline; (2) endoscopic therapies affecting neoplastic progression were performed during follow-up (Fig 1). Some manuscripts studied different biomarkers within the same population, these were considered as individual studies on the level of the individual biomarker.

### Data extraction

For each included study two independent authors extracted data according to a standardized data extraction form and assessed the quality of the eligible studies (S1 Standardized data extraction form). In case of disagreement consensus was reached by consulting a third author (MS). Odds ratio's (OR)s and 95% confidence intervals (CI)s of individual IHC biomarkers were extracted or estimated from the data. If ORs could not be extracted directly from the text or the tables, ORs were calculated indirectly by using the numbers of cases and controls with an aberrant versus a normal IHC biomarker expression from text, tables, or figures.

### Quality assessment

The quality aspects defined were: a difference at baseline between cases and controls of at least 10% (concerning baseline histology, age, sex, length of BE segment, and follow–up time), adjustments in the form of regression for differences of known predictors of progression (such as baseline histology, age, sex, and length of BE segment), exclusion of prevalent cases, control stainings, number of pathologists, pathologist agreement and pathologist blinding. These aspects were assessed and reported on but not used as exclusion criteria.

### Data synthesis and analyses

Meta-analysis were performed if at least two studies were available [27]. If multiple studies in a single analysis included the same patients, the oldest study was excluded. An inverse variance random-effect model was used. If data on multiple definitions of aberrant staining were available, definitions were chosen to resemble those from other included studies for that IHC biomarker. If only one study was available, definitions of the authors of that study were used. Pooled estimates of effect, in the form of ORs, were calculated and investigated for statistical heterogeneity by visual inspection and the I-squared test (I^2^) = [(Q-df)/Q]*100%, where Q was the chi squared statistic and df was its degree of freedom. Where possible, ORs adjusted for most factors were used in the analysis and unadjusted and adjusted ORs were pooled if necessary. Small study effects such as publication bias were assessed using a funnel plot.

### Sensitivity and subgroup analyses

Sensitivity analyses were performed in case of a large standard error (if small study effects were likely present as observed in the funnel plot), if no adjustments were made for known predictors of progression (such as sex, age, histology, i.e. non-dysplastic or LGD, and BE-length), and if only an abstract was available. We excluded individual studies from the most reliable analysis to evaluate the impact of single studies on pooled risk estimates and heterogeneity. Additional analyses were performed to assess if an IHC biomarker can be used as a predictor of neoplastic progression, independent of the presence of dysplasia. Therefore, all studies were summarized which included only non-dysplastic BE patients, only BE with LGD patients, or in which adjustments were made for histology type. Additionally, two subgroup analyses were performed including either non-dysplasic or LGD BE patients.

### Stringency of the definition for aberrant staining used and its interpretation

The stringency level of the definition for aberrant staining and its interpretation could lead to variation in the predictive ability of the IHC biomarker investigated. To investigate whether this effect might be present, the proportion of controls deemed positive was plotted against the OR of each study.

## Results

### Included studies

2081 records were retrieved, after removal of duplicates. After excluding 2050 records based on title and abstract, a total of 27 full text articles and four abstracts were assessed in detail (Fig 1). Of these, 19 full text articles and two abstracts were included in this review [28–49]. These articles contained a total of 36 studies.

#### Characteristics

A total of 36 studies were included, containing 2260 patients of which 425 cases, selected from a populations of more than 7.000 BE patients. The proportion of male patients ranged from 66% to 100%. Mean duration of follow-up varied from 11.3 months to 120 months. Most studies were retrospective case-control studies (n = 33), and three prospective cohort studies. One study defined BE as CLE without IM, other studies defined BE as CLE with IM (n = 23), or gave no definition (n = 12). Endpoint was EAC in six studies and either HGD or EAC in 30 studies. (Table 1).

**Table 1:** Characteristics of included studies.

Study	Marker	Design	Patients	Baseline	End-point	DEF BE	Antibody
Younes *et al*. 1997	p53	Retrospective case-control	25	LGD/IND	HGD/EAC	BE, IM+	BP-53-12 Bio Genex (m), (not mentioned)
Gimenez 1999	p53	Retrospective case-control	6	LGD	HGD/EAC	NA	Do-7, Dako, (m)(1:50)
Bani-Hani *et al*. 2000	p53	Retrospective nested case-control	52	IM, non-HGD	EAC	BE, IM+	DO-7-p53, Novocastra (m)(1:100)
Weston *et al*. 2001	p53	Prospective cohort	48	LGD	HGD/EAC	NA	Zymed, (m) (not mentioned)
Skacel *et al*. 2002	p53	Retrospective case-control	16	LGD	HGD/EAC	NA	Do-7, Dako, (m)(not mentioned)
Murray *et al*. 2006 a	p53	Retrospective nested case-control	197	IM	HGD/EAC	BE, IM+	DO-7-p53, Novocastra (m)(1:100)
Brown 2008	p53	Prospective cohort	276	IM, LGD	HGD/EAC	NA	not mentioned
Sikkema *et al*. 2009 a	p53	Retrospective nested case-control	42	IM, LGD	HGD/EAC	BE, IM+	Do-7, Dako, (m)(1:1000)
Bird-Lieberman *et al*.2012 a	p53	Retrospective nested case-control	356	IM,LGD	HGD/EAC	BE, IM+	DO7, Leica, (1:50)
Kastelein *et al*. 2012 a	p53	Case-control in prospective cohort	635	IM, LGD	HGD/EAC	BE, IM+	Do-7, Dako, (m)(1:25)
Wolf et al. 2014	p53	Retrospective nested case-control	279	IM, IND, LGD	EAC	NA	not mentioned
Davelaar *et al*. 2015	p53	Prospective cohort	91	IM, IND, LGD	HGD/EAC	BE, IM+	mix of DO-7 and PB53-12, Fisher scientific, (N/A)
Horvath et al. 2016	p53	Retrospective case-control	79	IND	HGD/EAC	NA	Do-7, Dako, (m)(1:20)
Bird-Lieberman *et al*.2012 c	AOL	Retrospective nested case-control	321	IM, LGD	HGD/EAC	BE, IM+	AOL 5ug biotinylated lectin, Tokyo chem. Indust.
Wolf et al. 2014	AOL	Retrospective nested case-control	252	IM, IND, LGD	EAC	NA	not mentioned
Pierre Lao-Sirieix *et al*. 2007	Cyclin A	Retrospective nested case-control	48	IM	EAC/HGD	BE, IM+	cyclin A, Novocastra (m)(1:20)
Bird-Lieberman *et al*.2012 b	Cyclin A	Retrospective nested case-control	323	IM, LGD	HGD/EAC	BE, IM+	Leica (m)(1:50)
Wolf et al. 2014	Cyclin A	Retrospective nested case-control	279	IM, IND, LGD	EAC	NA	not mentioned
Van Olphen et al. 2016	Cyclin A	Case-control in a prospective cohort	625	IM, LGD	HGD/EAC	BE, IM+	Leica (m)(1:200)
Bani-Hani *et al*. 2000	Cyclin D	Retrospective nested case-control	61	IM	EAC	BE, IM+	NCL-CYCLIN D1, Novocastra (m)(1:30)
Murray *et al*. 2006 b	Cyclin D	Retrospective nested case-control	197	IM	HGD/EAC	BE, IM+	NCL-L-CYCLIND1-GM, Novocastra, (m)(1:50)
Horvath et al. 2016	Cyclin D	Retrospective case-control	79	IND	HGD/EAC	NA	SP4, ThermoLabVision (m)(1:100)
Kastelein *et al*.*2013 b*	AMACR	Case-control in prospective cohort	631	IM, LGD	HGD/EAC	BE, IM+	clone 13H4, Thermo Scientific (m)(1:200)
Horvath et al. 2016	AMACR	Retrospective case-control	81	IND	HGD/EAC	NA	13H4, Zeta Corp (m)(1:100)
Lastraioli et al. 2006	hERG1	Retrospective cohort	23	IM	EAC	BE, IM+	hERG1 alexis corporation (p)(1:200)
Lastraioli et al. 2016	hERG1	Case-control	94	IM	EAC	NA	hERG1, Dival Toscana Srl (m)(1:200)
Sirieix et al. 2003	MCM2	Retrospective nested case-control	27	IM	HGD/EAC	BE, IM+	Hutchison, Cambridge, (m)(1:10)
Capello et al. 2005	CD1a	Retrospective case-control	166	CLE, IM negative	Dysplasia / EAC	BE, IM-	dako clone O10, (m)(1:50)
Murray *et al*. 2006 d	β-catenin	Retrospective nested case-control	194	IM	HGD/EAC	BE, IM+	G10153, Transduction Laboratories, (m)(1:100)
Murray *et al*. 2006 c	COX-2	Retrospective nested case-control	196	IM	HGD/EAC	BE, IM+	160112, Cayman Chemicals, (m)(1:250)
Sikkema *et al*. 2009 b	Ki-67	Retrospective case-control	42	IM	HGD/EAC	BE, IM+	Clone MIB-1, Dako (1:100)
Rossi *et al*. 2009	HER-2	Retrospective case-control	20	IM, LGD	HGD, EAC	NA	HercepTest^®^ kit, DAKOCytomation
Bird-Lieberman *et al*.2012 e	Sialyl Lewis	Retrospective nested case-control	356	IM, LGD	HGD/EAC	BE, IM+	BOND ready retrieval
Bird-Lieberman *et al*.2012 d	WGA	Retrospective nested case-control	331	IM, LGD	HGD/EAC	BE, IM+	Leica BOND-MAX
Bird-Lieberman *et al*.2012 f	Lewis	Retrospective nested case-control	350	IM, LGD	HGD/EAC	BE, IM+	CD15, BOND ready, retrieval H2 20 min Leica
Van Olphen *et al*. 2015	SOX2	Case-control in prospective cohort	635	IM, LGD	HGD/EAC	BE, IM+	AF2018, R&D Systems (p)(1:400)

#### Dilutions and definitions for aberrant staining used

For p53, the antibody DO-7 (Dako, Glostrup, Denmark) was frequently used with a dilution ranging from 1:20 to 1:1000. The definition for aberrant IHC staining was heterogeneous. Very intense staining was considered aberrant by all studies (being independent of the concentration used). However, intensity of staining was not a prerequisite for considering a staining pattern aberrant in seven studies[29–33, 37, 47]. Three more recent studies also considered a total absence of staining as aberrant [35, 36, 39]. For aspergillus oryzae lectin (AOL), one study calculated the OR for aberrant AOL IHC staining in 2 or 3 epithelial compartments versus 0 or 1 compartment [34]. Another study reported multiple ORs for aberrant AOL in 1, 2, or 3 versus 0 epithelial compartments [39]. The OR of aberrant AOL IHC staining in 2 or 3 versus 0 or 1 compartments was extracted from this second study and analyzed together with the data from the first study for the meta-analysis.

### Quality of studies

In 14 of the 36 studies there was at least a 10% difference in baseline histology between cases and controls. In these studies, around 32% of the cases had IND or LGD at baseline, versus 9% in the controls. In five studies an age difference at baseline of at least 5 years was found between cases and controls, in four of these studies the case group was older. In six studies at least 10% more males were included in the case groups, 96% males on average in the cases, versus 73% in controls. Information on length of the BE segment for both cases and controls was provided in 13 studies. In cases a longer BE segment was present; on average 5.9 cm versus 4.8 cm in the controls. In 17 studies the total follow-up time differed by at least 10% between case and control groups. On average, follow up time was 51 months versus 59 months for cases versus controls, respectively. Seven studies excluded possible prevalent cases [29, 32, 34, 47, 49]. Fourteen studies adjusted for known predictors of progression (S1 Table). 16 studies did not describe technical validation of the staining. IHC staining was scored by one observer in 15 studies, by two observers in 13 studies, and by three observers in three studies. Kappa values were mentioned in only eight studies. Whether slides were assessed in a blinded manner was not mentioned in six studies, all other studies reported the use of blinding (S1 Table).

### Meta-analyses

These were possible for p53, AOL, Cyclin A, Cyclin D, and alpha-methylacyl-CoA racemase (AMACR), which were studied 13, 2, 4, 3, and 2 times respectively. Of the 13 studies, two included patients from the same population, which resulted in exclusion of the older study in analyses for which both would have been eligible [32, 34]. The most frequent reasons for excluding articles were the absence of follow-up data and LGD being defined as neoplastic progression and end-point of the study (Fig 1). Biomarkers studied only once were MCM2, CD1a, β-catenin, COX2, Ki67, HER2, Sialyl Lewis, Wheat germ, Lewis, and SOX2. The same group published two studies on hERG1, both including patients from the same population[46, 48]. Therefore, both studies were individually included without summary in a meta-analysis.

### p53

**Fig 2:**
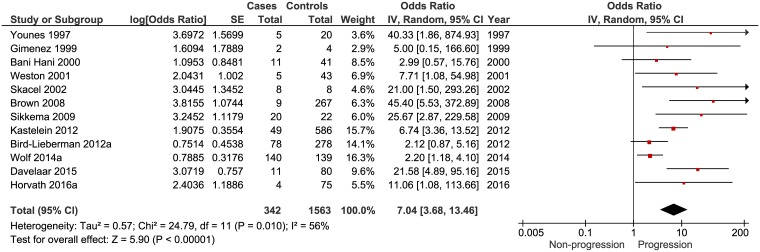
Forest plot of studies investigating p53 as a predictor of progression. Twelve studies were included.

A total of 12 studies were included in the meta-analysis. These contained 1905 patients, of which 342 cases. One study gave multiple ORs for various expression levels of p53 [34]. For this study, only the OR for intense overexpression of p53 staining was considered positive. Individual patient data of one study were converted in order to extract an adjusted OR [35]. The overall OR for neoplastic progression was 7.04 (95% CI 3.68 to 13.46) for patients with aberrant p53 expression (Table 2 and Fig 2). Aberrant p53 expression, detected in both non-dysplastic BE and LGD patients, was significantly associated with the development of HGD or EAC. Significant heterogeneity (I^2^ = 56%, P<0.010) was observed between the included studies, which can be considered a moderate amount of heterogeneity [50]. The 12 studies were plotted in a funnel plot which shows that small study effects can be present (S1 Fig). In order to reduce the influence of such effects a sensitivity analysis was performed, which excluded all studies with a standard error above one. Based on this criterion, five studies remained, containing 1413 patients and 289 cases. The overall OR for neoplastic progression was 4.15 (95% CI 1.96 to 8.81) in patients with an aberrant p53 expression. (Table 2). The use of a more stringent definition of aberrant staining may lead to loss of aberrant expression in cases, in controls, or in both. In order to investigate this, the proportion of controls deemed aberrant was plotted against the OR of each study (S2 Fig). Studies with a higher point estimate of the OR appeared to have had less positive non-progressors. The same is seen if this is analyzed in individual studies where multiple cut-offs for positivity are described[32, 34]. Using a more stringent cut off resulted in a higher OR. Further sub sensitivity analyses were performed excluding studies for which no adjusted ORs were available. Four studies remained, containing 1322 patients and 278 cases. The overall OR for neoplastic progression was 3.18 (95% CI 1.68 to 6.03) in patients with an aberrant p53 expression. (Table 2). Subsequently, individual studies were excluded from this analysis, and finally also studies presented as abstracts. These sensitivity analyses showed similar results with slightly lower point estimates compared to the main analysis. (Table 2). For three studies both unadjusted and adjusted ORs were available, and all three adjusted ORs had a lower point estimate compared to the unadjusted ones, in line with the outcome of our meta-analyses.

**Table 2:** Summary of meta-analyses of studies investigating p53 IHC as a predictor of neoplastic progression.

Analysis	Studies	Cases	Controls	OR	95% CI	I^2^	References
p53 (main)	12 (Fig 2)	342	1563	7.04	3.68–13.46	56%	[28–31, 33–39, 47]
p53 (excluded SE > 1)	5	289	1124	4.15	1.96–8.81	68%	[29, 34–36, 39]
p53 (also excluded unadjusted ORs)	4	278	1044	3.18	1.68–6.03	55%	[29, 34, 35, 39]
p53 (exclude individual study [29] from the above analysis)	3	267	1003	3.20	1.49–6.87	70%	[34, 35, 39]
p53 (exclude individual study [34] from the above analysis)	3	200	766	3.64	1.57–8.41	64%	[29, 35, 39]
p53 (exclude individual study [35] from the above analysis)	3	229	458	2.23	1.37–3.64	0%	[29, 34, 39]
p53 (exclude individual study [39] from the above analysis)	3	138	905	3.78	1.65–8.68	52%	[29, 34, 35]
p53 (also excluded abstracts)	3	138	905	3.78	1.65–8.68	52%	[29, 34, 35]
p53 (only ORs stratified for histology)	6	282	1058	3.86	2.03–7.33	46%	[30, 31, 34, 35, 37, 39]
p53 (only non-dysplastic BE)	2	61	659	6.12	2.99–12.52	0%	[32, 35]
p53 (only LGD)	4	37	145	8.64	3.62–20.62	0%	[30, 31, 35, 37]

#### p53 as independent predictor of neoplastic progression

For this analysis studies that did not adjust for histology at baseline were excluded. This led to the inclusion of six studies. These studies contained a total of 1340 BE patients, of which 282 cases. The overall OR, for aberrant p53 IHC on neoplastic progression, after stratification for histology, was 3.86 (95% CI 2.03 to 7.33). (Table 2).

#### p53 in non-dysplastic Barrett's oesophagus

Two studies were included for this analysis. These contained a total of 720 BE patients, of which 61 cases. Individual patient data of one study was re-analyzed to provide an OR for non-dysplastic BE patients only [35]. The overall OR for neoplastic progression to HGD or EAC in non-dysplastic BE patients was 6.12 (95% CI 2.99 to 12.52). (Table 2).

#### p53 in low-grade dysplasia Barrett

For this analysis four studies were included. These contained a total of 182 BE patients, of which 37 cases. One study was re-analyzed to provide an OR for the LGD subgroup only [35]. The overall OR for neoplastic progression to HGD or EAC was 8.64 (95% CI 3.62 to 20.62). (Table 2).

### AOL

Two studies were included in this meta-analysis. These contained 573 BE patients, of which 204 cases. The overall OR for neoplastic progression in BE patients with an aberrant AOL staining in 2 or 3 compartments, versus 0 or 1 compartments of the tissue was 3.04 (95% CI 2.05 to 4.49) (Table 3 and S3 Fig). Results of the two studies were consistent in their findings (I^2^ = 0%, P = 0.85).

**Table 3:** Summary of meta-analyses of studies investigating IHC biomarkers other than p53 as a predictor of neoplastic progression.

Analysis	Studies	Cases	Controls	OR	95% CI	I2
AOL	2 (S3 Fig)	204	369	3.04	2.04–4.49	0%
Cyclin A	4 (S3 Fig)	285	990	1.90	0.85–4.22	76%
Cyclin D	3 (S3 Fig)	50	287	1.01	0.14–7.03	80%
AMACR	2 (S3 Fig)	53	659	4.07	0.66–25.12	53%

### CYCLIN A

Four studies were included in this meta-analysis. These contained 1275 patients, of which 285 cases. The overall OR for neoplastic progression in BE patients with cyclin A positivity was 1.90 (95% CI 0.85 to 4.22) (Table 3 and S3 Fig). Results of the three studies were inconsistent in their findings (I^2^ = 76%, P = 0.005).

### CYCLIN D

Three studies were included in this meta-analysis. These contained 337 patients, of which 50 cases. The overall OR for neoplastic progression in BE patients with cyclin D positivity was 1.01 (95% CI 0.14 to 7.03) (Table 3 and S3 Fig). Results of the two studies were inconsistent in their findings (I^2^ = 80%, P = 0.007).

### Alpha-methylacyl-CoA racemase

Two studies were included in this meta-analysis. These contained 712 patients, of which 53 cases. The overall OR for neoplastic progression in BE patients with alpha-methylacyl-CoA racemase positivity was 4.07 (95% CI 0.66 to 25.12) (Table 3 and S3 Fig). Results of the two studies were moderately consistent in their findings (I^2^ = 53%, P = 0.14).

### Studies on other IHC biomarkers

The following IHC biomarkers were investigated only once: β-catenin, CD1a, COX2, HER2, Ki67, Lewis, Mcm2, Sialyl Lewis, SOX2, and WGA. The same group published two studies on hERG1, both including patients from the same population[46, 48]. Therefore, both studies were individually included without summary in a meta-analysis. In the CD1a study CLE without IM was used as baseline histology [45]. When considering study size and point estimate, CD1a, SOX2, and hERG1 appeared most promising. (S4 Fig).

## Discussion

This is the first systematic review and meta-analysis to assess if IHC biomarkers can be used as an independent predictor for neoplastic progression in BE surveillance. Sixteen biomarkers have been investigated in this setting, of which five biomarkers have been investigated more than once. The meta-analysis showed that aberrant p53 expression was associated with a significantly increased risk of neoplastic progression. Moreover, aberrant p53 expression predicted neoplastic progression in both non-dysplastic BE patients and BE patients with LGD. Of the other four IHC biomarkers, AOL appeared to be most promising in predicting neoplastic progression, whereas Cyclin A, Cyclin D, and alpha-methylacyl-CoA racemase are still of limited value.

Current use of p53 IHC in BE patients differs in international guidelines. The guideline of the British Society of Gastroenterology recommends the addition of p53 IHC staining for the pathological assessment of BE to improve the diagnostic reproducibility of dysplasia [5]. While the American Gastroenterological Association guideline states that: “data supporting the use of biomarkers to confirm the histologic diagnosis of dysplasia must be considered preliminary [51]. No guideline has yet adopted the use of IHC biomarkers to predict neoplastic progression. Two large population based studies confirmed that patients with LGD have an approximately 5 times higher risk of neoplastic progression compared to patients without LGD [15, 18]. Our meta-analysis is the first to show that BE patients, independent of the presence of LGD, with aberrant p53 IHC have a similar increased risk to develop cancer compared to patients with LGD. A recent publication claims to have investigated the predictive ability of immunohistochemical biomarkers [52]. However, they reported on samples either obtained from a resection specimen or from cases and controls without follow-up. Therefore, based on their current dataset, their current conclusion, i.e. that p53 overexpression predicts malignant progression, is not justified [53].

Although routine p53 IHC will incur higher cost than histological assessment alone, application of this marker has the potential to reduce the overall costs related to BE surveillance by improved risk stratification using expression of p53 IHC in combination with other predictors of progression, such as histology, sex, age, and length of the BE segment. Better risk stratification could result in both earlier detection of lesions in patients at risk, and a reduction in endoscopic and pathology recources for patients that will never develop progression. The disparity in ORs of neoplastic progression found in the various studies may be explained by differences in staining methods, including antibodies used, antigen retrieval methods, definitions, and interpretations of aberrant staining used. Therefore, special consideration should be given to the protocol of staining and the definition and interpretation used for aberrant expression. Some studies did not consider loss of p53 staining aberrant, which might have contributed to the protocol being less predictive compared to other studies. By using a more stringent definition of aberrant expression, cases appeared to remain p53 aberrant, while controls were not considered aberrant (S2 Fig). Therefore, the use of more stringent definitions and interpretations for aberrant staining appears to lead to a higher predictive ability of p53 IHC.

The strength of this paper is the focus on IHC biomarkers as a relatively easy applicable tool to improve risk stratification in BE surveillance. Additionally, we performed a broad search, and the extraction of ORs from text, tables and figures resulted in the inclusion of quite a large number of studies. The inclusion of abstracts results in an up to date overview of this field. Because meta-analysis is the synthesis technique that is most transparent and most likely valid also with small amount of studies included, some of the meta-analysis were performed with only two studies, as no more studies were available. [27] This study also has its limitations, such as the confinement to English language publications, the apparent presence of publication bias, differences in baseline comparability within studies, and the various adjustments made for these baseline differences. Therefore, we performed sensitivity analyses of the p53 meta-analyses, these show that the point estimate of the OR decreased from 7.04 to 3.18 when we accounted for these limitations. Because aberrant p53 IHC co-occurs with LGD, separate analyses were performed in which we stratified for dysplastic and non-dysplastic patients. These analyses show that aberrant p53 expression is an independent prognostic factor for neoplastic progression.

In conclusion, we show that sixteen IHC biomarkers in BE surveillance have been studied. Aberrant p53 expression is the most studied IHC biomarker and associated with a significantly increased risk to develop HGD or EAC, this association was independent of the presence of LGD. Consensus amongst pathologists concerning the appropriate staining method, definition, and interpretation of aberrant p53 expression is currently low, and more consensus is required. Other promising biomarkers such as AOL need further investigation.

## Supporting information

S1 Fig Funnel plot of all studies investigating p53 IHC as a predictor of progression. The exact patient numbers and the SE of these studies can be found in Fig 2.
(EPS)

S2 Fig Stringency of the definition and interpretation of aberrant p53 IHC. A more stringent definition of aberrant staining, and interpretation of that definition, may lead to loss of aberrant expression in cases, in controls, or in both. In order to investigate this, the proportion of controls deemed aberrant was plotted against the OR of each study with a standard error below 1. The use of a more stringent definition and interpretation for aberrant p53 staining appeared to result in a bigger reduction in the number of controls considered to have aberrant staining, compared to cases. Thus, by applying a more stringent definition and interpretation, the predictive value of p53 for neoplastic progression appears to increase. Formal statistical tests were not performed due to the limited number of data points and the post hoc nature of this analysis.
(EPS)

S3 Fig Forest plot of all studies investigating AOL, Cyclin A, Cyclin D, and AMACR as a predictor of progression. (A) Two studies were included in the forest plot for AOL. (B) Four studies were included in the forest plot for Cyclin A. (C) Three studies were included in the forest plot for Cyclin D. (D) Two studies were included in the forest plot for AMACR.
(TIF)

S4 Fig Forest plot, without meta-analysis, of all studies investigating IHC biomarkers which have been studied only once. 12 studies were included in this forest plot.
(EPS)

S1 Table Additional characteristics of included studies. (DOCX)

S1 Search (DOCX)

S1 Standardized data extraction form (DOCX)

S1 MOOSE checklist (DOCX)

S1 PRISMA checklist (DOCX)
