# Supplementary material for: Use of immunohistochemical biomarkers as independent predictor of neoplastic progression in Barrett's oesophagus surveillance: A systematic review and meta-analysis
Source: PLoS One. 2017 Oct 23;12(10):e0186305. doi: 10.1371/journal.pone.0186305 (PMC5653304; doi:10.1371/journal.pone.0186305)
Supplement: S1 Table — (DOCX) [file pone.0186305.s005.docx]

**Supplementary table 1. Additional characteristics of included studies.**

| **Study** | **Marker** | **Data adjusted for:** | **follow up time (mean in months unless otherwise specified)** | **age (mean in years)** | **proportion male (%)** | **# pathologists** | **blind assesment** |
| --- | --- | --- | --- | --- | --- | --- | --- |
| Younes *et al.* 1997 | p53 | Unadjusted | 22 months for p53+, 25 months for p53- | not mentioned | not mentioned | 1 | Yes |
| Gimenez 1999 | p53 | Unadjusted | 84 cases and 40.8 controls | 62.3 all patients | 66% all patients | 1 | Yes |
| Bani-Hani *et al.* 2000 | p53 | sex, age, follow-up, BE length, # of biospy specimens | 52.0 cases and 56.5 controls | 60.3 cases and 61.5 controls | 92% cases and 94% controls | 1 | Yes |
| Weston *et al.* 2001 | p53 | Unadjusted | 41.2 all patients | 61.9 all patients | 100% all patients | 3 | N/A |
| Skacel *et al.* 2002 | p53 | Unadjusted | 11.3 cases and 35.4 controls | 62 cases and 67.4 controls | 100% cases and 62.5% controls | 3 | Yes |
| Murray *et al.* 2006 a | p53 | pathlogy laboratory | 27.6 cases and 46.8 controls | 68 cases and 67.1 controls | 77.1% all patients | 2 | Yes |
| Brown 2008 | p53 | Unadjusted | 41 months all patients | not mentioned | not mentioned | N/A | N/A |
| Sikkema *et al.* 2009 a | p53 | Unadjusted | 82.8 cases and 94.8 controls | 58.8 cases and 56.2 controls | 89% cases and 74% controls | 2 | Yes |
| Bird-Lieberman *et al.*2012 a | p53 | age, sex, year of diagnosis, histology | 80.4 all patients | 63.8 cases and 63.8 controls | 75% cases and 75% controls | 10% had 2^nd^ observer | Yes |
| Kastelein *et al.* 2012 a | p53 | age, sex, BE length, and esophagistis, after re-analysis adjusted for age, sex, BE length, and histology | median 81.6 controls and 36 cases | median 60 controls and 65 | 82% cases and 73% controls | 2 | Yes |
| Wolf et al. 2014 | p53 | age, BE length, time in study, and histology | 46.8 cases and 38.4 controls | 60 cases and 60 controls | 82% cases and 81% non-cases | N/A | Yes |
| Davelaar *et al.* 2015 | p53 | Age, sex, BE length, after extracting the OR no adjustment could be made. | median 71 all patients | 62 all patients | 85% all patients | 1 | Yes |
| Horvath et al. 2016 | P53 | Unadjusted | Median 59 months | 60.5 cases and 63 controls | 100% cases and 76% controls | 1 | Yes |
| Bird-Lieberman *et al.*2012 c | AOL | age, sex, year of diagnosis, higtology | 80.4 all patients | 63.8 cases and 63.8 controls | 75% cases and 75% controls | 10% had 2^nd^ observer | Yes |
| Wolf et al. 2014 | AOL | Unadjusted | 46.8 cases and 38.4 controls | 60 cases and 60 controls | 82% cases and 81% non-cases | N/A | Yes |
| Pierre Lao-Sirieix *et al.* 2007 | Cyclin A | Age, gender, length of follow-up | 78 cases and 66 controls | 62.2 cases and 56.7 controls | 87.5% cases and 72.2% controls | 1 | Yes |
| Bird-Lieberman *et al.*2012 b | Cyclin A | age, sex, year of diagnosis, higtology | 80.4 all patients | 63.8 cases and 63.8 controls | 75% cases and 75% controls | 10% had 2^nd^ observer | Yes |
| Wolf et al. 2014 | Cyclin A | Unadjusted | 46.8 cases and 38.4 controls | 60 cases and 60 controls | 82% cases and 81% non-cases | N/A | Yes |
| Van Olphen et al. 2016 | Cyclin A | age, sex, BE length, and histology | Median 80.4 months | 65 cases and 60 controls | 82% cases and 73% controls | 2 | Yes |
| Bani-Hani *et al.* 2000 | Cyclin D | sex, age, follow-up, BE length, # of biospy specimens | 52.0 cases and 56.5 controls | 60.3 cases and 61.5 controls | 92% cases and 94% controls | 1 | Yes |
| Murray *et al.* 2006 b | Cyclin D | pathlogy laboratory | 27.6 cases and 46.8 controls | 68 cases and 67.1 controls | 77.1% all patients | 2 | Yes |
| Horvath et al. 2016 | Cyclin D | Unadjusted | Median 59 months | 60.5 cases and 63 controls | 100% cases and 76% controls | 1 | Yes |
| Kastelein *et al.2013 b* | AMACR | Unadjusted | median 36 cases and 81.6 controls | median 60 controls and 65 cases | 82% cases and 73% controls | 2 | Yes |
| Horvath et al. 2016 | AMACR | Unadjusted | Median 59 months | 60.5 cases and 63 controls | 100% cases and 76% controls | 1 | Yes |
| Sirieix et al. 2003 | MCM2 | Unadjusted | 72 cases and controls 60 | not mentioned | not mentioned | N/A | N/A |
| Capello et al. 2005 | CD1a | Unadjusted | 12-36 all patients | not mentioned | not mentioned | 2 | N/A |
| Murray *et al.* 2006 d | β-catenin | pathlogy laboratory | 27.6 cases and 46.8 controls | 68 cases and 67.1 controls | 77.1% all patients | 2 | Yes |
| Lastraioli et al. 2006 | hERG1 | Unadjusted | at least 5 years | not mentioned | not mentioned | 2 | N/A |
| Murray *et al.* 2006 c | COX-2 | pathlogy laboratory | 27.6 cases and 46.8 controls | 68 cases and 67.1 controls | 77.1% all patients | 2 | Yes |
| Sikkema *et al.* 2009 b | Ki-67 | Unadjusted | 82.8 cases and 94.8 controls | 58.8 cases and 56.2 controls | 89% cases and 74% controls | 2 | Yes |
| Rossi *et al.* 2009 | HER-2 | Unadjusted | 45.8 cases and 54.7 controls | 63 years | 76% all patients | 2 | N/A |
| Bird-Lieberman *et al.*2012 e | Sialyl Lewis | age, sex, year of diagnosis, higtology | 80.4 all patients | 63.8 cases and 63.8 controls | 75% cases and 75% controls | 10% had 2^nd^ observer | Yes |
| Bird-Lieberman *et al.*2012 d | wheat germ agglutinin | age, sex, year of diagnosis, higtology | 80.4 all patients | 63.8 cases and 63.8 controls | 75% cases and 75% controls | 10% had 2^nd^ observer | Yes |
| Bird-Lieberman *et al.*2012 f | Lewis | age, sex, year of diagnosis, higtology | 80.4 all patients | 63.8 cases and 63.8 controls | 75% cases and 75% controls | 10% had 2^nd^ observer | Yes |
| Van Olphen *et al.* 2015 | SOX2 | Unadjusted | median 39.6 cases and 78 controls | median 60 controls and 65 cases | 82% cases and 73% controls | 2 | Yes |
| Lastraioli et al. 2016 | hERG1 | age and sex | At least 120 | not mentioned | not mentioned | 3 | N/A |
