# Supplementary material for: Use of immunohistochemical biomarkers as independent predictor of neoplastic progression in Barrett's oesophagus surveillance: A systematic review and meta-analysis
Source: PLoS One. 2017 Oct 23;12(10):e0186305. doi: 10.1371/journal.pone.0186305 (PMC5653304; doi:10.1371/journal.pone.0186305)
Supplement: S1 Search — (DOCX) [file pone.0186305.s006.docx]

**EMBASE search.**

(immunohistochemistry/exp OR Immunocytochemistry/exp OR histochemistry/de OR 'biological marker'/exp OR 'disease marker'/de OR 'tumor marker'/de OR 'molecular marker'/de OR marker/de OR staining/de OR 'antibody labeling'/exp OR (immunohistochem* OR Immunocytochemistr* OR Immunohistocytochemis* OR immunostain* OR stain* OR histochemist* OR Histocytochemist* OR marker* OR biomarker* OR (marking NEAR/3 agent*) OR ((antibod* OR immun*) NEAR/3 label*) OR immunolabel*):ab,ti) AND ('oncogenesis and malignant transformation'/exp OR 'cancer risk'/de OR 'disease course'/de OR ('esophageal adenocarcinoma'/exp AND 'risk assessment'/exp) OR (((malign* OR metasta* OR cancer* OR adenocarcinom* OR tumo* OR ac OR eac OR high-grade OR hgd OR neoplas*) NEAR/10 (potential* OR transformat* OR predict* OR progress* OR develop* OR risk OR growth OR progress*)) OR (risk NEAR/3 progress*) OR carcinogene* OR oncogene* OR (tumo* NEAR/3 promot*)):ab,ti) AND ('Barrett esophagus'/exp OR (Barret*):ab,ti) AND [english]/lim NOT ([animals]/lim NOT [humans]/lim) NOT ([Conference Abstract]/lim OR [Letter]/lim OR [Note]/lim OR [Editorial]/lim)

**MEDLINE search.**

(exp immunohistochemistry/ OR Histocytochemistry/ OR "Biological Markers"/ OR "disease marker"/ OR exp "Tumor Markers, Biological"/ OR exp "Staining and Labeling"/ OR (immunohistochem* OR Immunocytochemistr* OR Immunohistocytochemis* OR immunostain* OR stain* OR histochemist* OR Histocytochemist* OR marker* OR biomarker* OR (marking ADJ3 agent*) OR ((antibod* OR immun*) ADJ3 label*) OR immunolabel*).ab,ti.) AND (exp "Carcinogenesis"/ OR "Disease Progression"/ OR ("Esophageal Neoplasms"/ AND "Risk Assessment"/) OR (((malign* OR metasta* OR cancer* OR adenocarcinom* OR tumo* OR ac OR eac OR high-grade OR hgd OR neoplas*) ADJ10 (potential* OR transformat* OR predict* OR progress* OR develop* OR risk OR growth OR progress*)) OR (risk ADJ3 progress*) OR carcinogene* OR oncogene* OR (tumo* ADJ3 promot*)).ab,ti.) AND ("Barrett Esophagus"/ OR (Barret*).ab,ti.) AND english.la. NOT (exp animals/ NOT humans/) NOT (letter OR news OR comment OR editorial OR congresses OR abstracts).pt.

**Web of Science search.**

((immunohistochem* OR Immunocytochemistr* OR Immunohistocytochemis* OR immunostain* OR stain* OR histochemist* OR Histocytochemist* OR marker* OR biomarker* OR (marking NEAR/3 agent*) OR ((antibod* OR immun*) NEAR/3 label*) OR immunolabel*):ab,ti) AND ((((malign* OR metasta* OR cancer* OR adenocarcinom* OR tumo* OR ac OR eac OR high-grade OR hgd OR neoplas*) NEAR/10 (potential* OR transformat* OR predict* OR progress* OR develop* OR risk OR growth OR progress*)) OR (risk NEAR/3 progress*) OR carcinogene* OR oncogene* OR (tumo* NEAR/3 promot*)):ab,ti) AND ((Barret*):ab,ti)

**CENTRAL search.**

TS=(((immunohistochem* OR Immunocytochemistr* OR Immunohistocytochemis* OR immunostain* OR stain* OR histochemist* OR Histocytochemist* OR marker* OR biomarker* OR (marking NEAR/2 agent*) OR ((antibod* OR immun*) NEAR/2 label*) OR immunolabel*)) AND ((((malign* OR metasta* OR cancer* OR adenocarcinom* OR tumo* OR ac OR eac OR high-grade OR hgd OR neoplas*) NEAR/10 (potential* OR transformat* OR predict* OR progress* OR develop* OR risk OR growth OR progress*)) OR (risk NEAR/2 progress*) OR carcinogene* OR oncogene* OR (tumo* NEAR/2 promot*))) AND ((Barret*)) NOT ((animal* OR rat OR rats OR mouse OR mice OR murine) NOT (human* OR patient*))) AND dt=(article)

**Pubmed publisher search.**

(immunohistochemistry[mh] OR Histocytochemistry[mh] OR "Biological Markers"[mh] OR "disease marker"[mh] OR "Tumor Markers, Biological"[mh] OR "Staining and Labeling"[mh] OR (immunohistochem*[tiab] OR Immunocytochemistr*[tiab] OR Immunohistocytochemis*[tiab] OR immunostain*[tiab] OR stain*[tiab] OR histochemist*[tiab] OR Histocytochemist*[tiab] OR marker*[tiab] OR biomarker*[tiab] OR (marking AND agent*[tiab]) OR ((antibod*[tiab] OR immunol*[tiab] OR immuni*[tiab]) AND label*[tiab]) OR immunolabel*[tiab])) AND ("Carcinogenesis"[mh] OR "Disease Progression"[mh] OR ("Esophageal Neoplasms"[mh] AND "Risk Assessment"[mh]) OR (((malign*[tiab] OR metasta*[tiab] OR cancer*[tiab] OR adenocarcinom*[tiab] OR tumor*[tiab] OR tumour*[tiab] OR ac OR eac OR high-grade OR hgd OR neoplas*[tiab]) AND0 (potential*[tiab] OR transformat*[tiab] OR predict*[tiab] OR progress*[tiab] OR develop*[tiab] OR risk OR growth OR progress*[tiab])) OR (risk AND progress*[tiab]) OR carcinogene*[tiab] OR oncogene*[tiab] OR ((tumor*[tiab] OR tumour*[tiab]) AND promot*[tiab]))) AND ("Barrett Esophagus"[mh] OR (Barret*[tiab])) AND english[la] NOT (animals[mh] NOT humans[mh]) NOT (letter[pt] OR news[pt] OR comment[pt] OR editorial[pt] OR congresses[pt] OR abstracts[pt]) AND publisher[sb]

**Google scholar search.**

immunohistochemistry|Immunocytochemistristry|immunostaining|histochemististry|immunolabeling "Barrett esophagus|oesophagus" malignant|metastatic|cancer|tumor potential|transformation|progression|progression|carcinogenesis|oncogenesis
