# Supplementary material for: Use of immunohistochemical biomarkers as independent predictor of neoplastic progression in Barrett's oesophagus surveillance: A systematic review and meta-analysis
Source: PLoS One. 2017 Oct 23;12(10):e0186305. doi: 10.1371/journal.pone.0186305 (PMC5653304; doi:10.1371/journal.pone.0186305)
Supplement: S1 Standardized data extraction form — (DOCX) [file pone.0186305.s007.docx]

**Standardized data extraction form.**

A standardized data extraction form was used, which contained the following items:

- General information: title, authors, source, contact address, country, published/unpublished, full paper / abstract, language, and year of publication.
- Study design.
- Quality assessment: a difference at baseline between cases and controls of at least 10% (concerning baseline histology, age, sex, length of BE segment, and follow –up time), adjustments in the form of regression for differences of known predictors of progression (such as baseline histology, age, sex, and length of BE segment), exclusion of prevalent cases, control stainings, number of pathologists, pathologist agreement and pathologist blinding.
- Patients: baseline histology, end-point histology, definition of BE used, age of the patients, proportion of male patients.
- Staining characteristics: IHC biomarker studied, antibody used and dilution, cutoff value of IHC biomarker expression used, positive/negative control used.
- Outcomes: Numbers of IHC biomarker positive cases and controls and IHC biomarker negative cases and controls. ORs and the factors that were adjusted for.
