# Supplementary figures and images for: Use of immunohistochemical biomarkers as independent predictor of neoplastic progression in Barrett's oesophagus surveillance: A systematic review and meta-analysis
Source: PLoS One. 2017 Oct 23;12(10):e0186305. doi: 10.1371/journal.pone.0186305 (PMC5653304; doi:10.1371/journal.pone.0186305)

Meta-analysis of Observational Studies in Epidemiology (MOOSE) checklist:


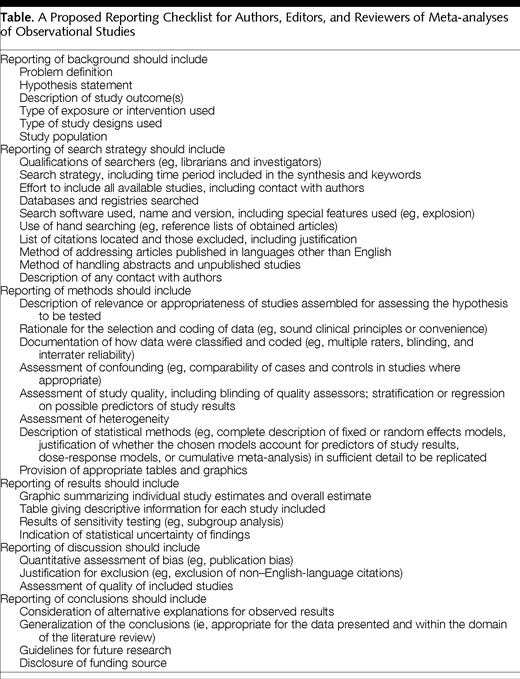

Supplement: S1 MOOSE checklist — (DOCX) [file pone.0186305.s008.docx]
